# Characterization of a novel splicing mutation in *UNC13D* gene through amplicon sequencing: a case report on HLH

**DOI:** 10.1186/s12881-017-0489-1

**Published:** 2017-11-21

**Authors:** Dongling Liu, Xijiang Hu, Xiwen Jiang, Bo Gao, Cheng Wan, Changying Chen

**Affiliations:** 1 School of Nursing, Zhengzhou University, Zhengzhou, Henan 450052 China; 2 Wuhan Children’s Hospital (Wuhan Maternal and Child Healthcare Hospital), Tongji Medical College, Huazhong University of Science and Technology, Wuhan, Hubei 430016 China; 3 DaAn Gene Co., Ltd. Of Sun Yat-sen University, The Medicine and Biological Engineering Technology Research Center of the Ministry of Health, Guangzhou, Guangdong China; 4 Department of Laboratory Medicine, Taihe Hospital, Hubei University of Medicine, Shiyan, Hubei China; 5 Department of Biomedical Engineering, College of Engineering, Peking University, Beijing, 100871 China

**Keywords:** HLH, *UNC13D*, Splicing mutation, Amplicon sequencing, Genetic analysis

## Abstract

**Background:**

Hemophagocytic lymphohistiocytosis (HLH) is a rare but fatal disease caused by uncontrolled proliferation of activated lymphocytes and macrophages. Six genes including *SH2D1A, PRF1, UNC13D, STX11, STXBP2* and *XIAP* were reported as causative genes in most cases.

**Case presentation:**

Here we report a novel splicing mutation in UNC13D gene, which was identified in an 18-year-old female. Patient was diagnosed as HLH base on HLH-2004 guidelines, no history of inherited diseases was revealed in this family, parents were healthy and non-consanguineous. Splenomegaly and hemophagocytosis in bone marrow were observed in clinical examination. Amplicon sequencing for the whole coding region of 6 HLH-related genes was performed on Ion S5XL genetic analyzer. In all, four heterozygous mutations were detected, including 2 nonpathogenic SNPs (*PRF1*:c.900C > T, *STX11*:c.*70G > A) and 2 splicing mutations in *UNC13D* gene (*UNC13D*:c.1299 + 1G > A and *UNC13D*:c.2709 + 1G > A), both of which were predicted to be potentially pathogenic by human splicing finder (HSF3) tool. The result was confirmed by two-generation pedigree analysis base on sanger sequencing.

**Conclusions:**

Two compound heterozygous splicing mutations in *UNC13D* gene were identified and considered to be potential pathogenesis in a female patient of HLH. The mutation *UNC13D*:c.1299 + 1G > A was reported in HLH for the first time. The inheritance mode and source of the mutation in the proband was examined by family analysis. Our data suggest that further studies of the spectrum of HLH-related mutations in China are warranted.

**Electronic supplementary material:**

The online version of this article (10.1186/s12881-017-0489-1) contains supplementary material, which is available to authorized users.

## Background

Hemophagocytic lymphohistiocytosis (HLH) was first reported by Farquhar et al. at 1952 [1]. It is a rare but fatal disease caused by uncontrolled proliferation of activated lymphocytes and macrophages [2], with the mortality rate ranging from 22 to 60% [3]. The familial HLH (FHL) are usually diagnosed in childhood, while secondary HLH can occur at any age [4]. However, the true epidemiology of HLH is difficult to access due to the limited epidemiologic data. It is believed that the incidence of HLH is underestimated, as it is not pathologically evident until autopsy, and often diagnosed as other disease with similar symptoms [5].

Rapid definitive diagnosis and appropriate treatment is necessary for life-saving and improved prognosis for patients of HLH. Current clinical and laboratory criteria help significantly in accurate diagnosis, but most of them are not time-saving. The sensitivity and specificity are not good enough either. Hence, the gene mutation analysis based on amplicon sequencing could be an essential tool for the definitive diagnosis of HLH.

Here, high throughput amplicon sequencing was conducted in an 18-year-old patient, to detect mutations in *SH2D1A, PRF1, UNC13D, STX11, STXBP2* and *XIAP* gene, which had been reported as the most common cause of HLH [6–8]. Two heterozygous splicing mutations were identified, one of which had been reported, and the other is novel, both of them were confirmed by Sanger sequencing. The result suggests that amplicon sequencing is an efficient and accurate tool in diagnosis of HLH, and it could be helpful for improving the understanding of this disease.

## Case presentation

The study was approved by the institute ethics committee on the use of human subjects in Wuhan Children’s Hospital. The CARE guidelines were followed in this case. Informed consent from the patient and parents was obtained before collecting blood samples. An 18-year-old female with HLH was diagnosed based on blood analysis and genetic detection. The relative mutations were two heterozygous splicing mutations in *UNC13D* gene (c.1299-1G > A in exon 15 and c.2709 + 1G > A in intron 28) and both were validated by Sanger sequencing. This patient and her parents are all Han Chinese from Hubei province of China.

**Fig. 1:**
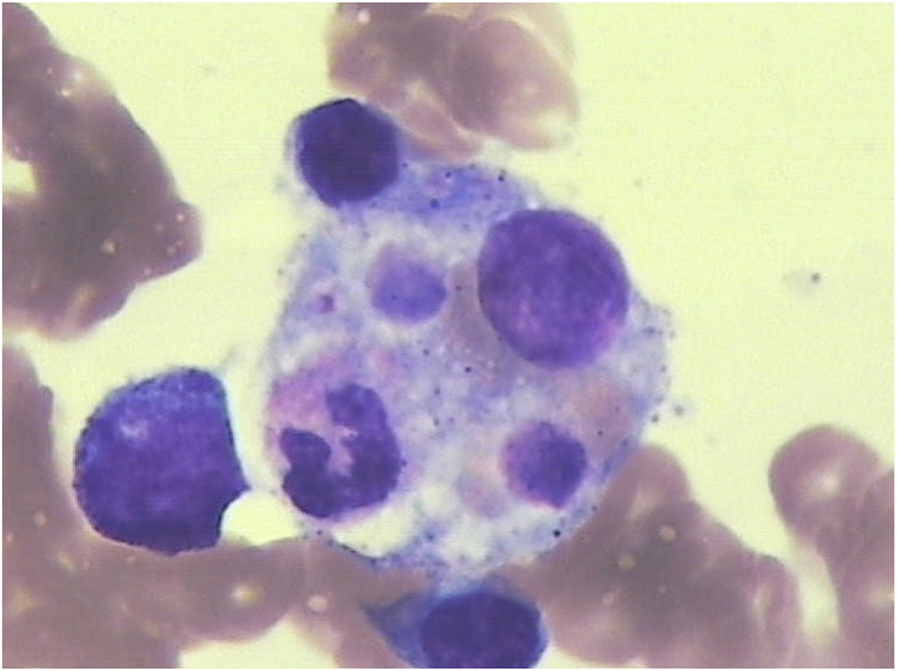
The bone marrow examination. Phagocytosis could be clearly observed in the boon marrow. No evidence of malignancy was observed

The 18-year-old female patient had fever for more than 2 days with unknown origin when she was admitted to Wuhan Children’s Hospital. No medicine had been taken before admission. The temperature was measured to 40.0 °C on regular examination. Ultrasound examination showed a slight hepatomegaly. Liver function test resulted in alanine aminotransferase (ALT) of 379 IU/L, aspartate transaminase (AST) of 158 IU/L, and triglyceride (TG) of 8 mmol/L. Blood analysis was conducted at clinical laboratory of Wuhan Children’s Hospital. Platelets (PLT), white blood cell (WBC) and red blood cell (RBC) were measured to be 50 × 10^9^/L, 3.5 × 10^9^/L and 2.8 × 10^12^/L, respectively. Low NK-cell activity (4.75%) and low plasma albumin (19.2 g/L) were also observed. The bone marrow examination suggested hemophagocytosis with no evidence of malignancy (Fig. 1). The results of the blood analysis and clinical features suggested the diagnosis of HLH based on HLH-2004 guidelines [9]. A two-generation pedigree including the patient and her parents was performed.

Genomic DNA was purified from peripheral blood mononuclear cells (PBMC) with QIAamp Blood Kit (Qiagen, Hilden, Germany) according to manufacturer’s protocol. Multiple PCR primers were designed for 6 HLH-related genes (*SH2D1A, PRF1, UNC13D, STX11, STXBP2* and *XIAP*) using Ion AmpliSeq™ Designer (https://www.ampliseq.com). All 64 exons of these genes were covered by 198 amplicons, including the whole coding regions and splicing sites as well. Product of multiple PCR was then processed and sequenced with S5XL genetic analyzer (Applied Biosystems^®^, Life Technologies, Grand Island, NY, USA). VariantCaller V1.0 was used to identify mutations including point mutation, insertion, deletion, structural variation, and so on.

Average base coverage depth of the target region was 1133-fold, over 96.94% of total base was sequenced more than 100-fold. The uniformity of coverage was 93.84%, suggesting a good performance of amplicon generating and sequencing. Only 4 SNPs were identified in the whole target region, each of them was heterozygous (Table 1). According to current database and clinical reports, one was known mutation recorded in the NCBI refSNP database (ID: rs3734228) and no clinical symptoms had been reported to be associated with it. Two were reported by Zhizhuo H, et al. in 2012 [8], one of which was a synonymous mutation (ID: rs885822) and was considered to be benign, another one was a splicing mutation located in intron 28 of *UNC13D* gene. The last SNP was novel, which was also a splicing mutation located in exon 15 of *UNC13D* gene.

**Table 1:** Information of mutations detected in patient with amplicon sequencing

Gene-exon	Position	Type	Zygosity	Reference	Variant	Frequency of variant	Coverage	^a^ID in dbSNP	Allele Frequency in gnomAD
PRF1-Exon3	c.900C > T	synonymous mutation	Heterozygous	G	A	50.3	500	rs885822	0.6403
UNC13D-exon15	c.1299-1G > A	Splicing	Heterozygous	C	T	49.3	800	novel	Unknown
UNC13D-intron28	c.2709 + 1G > A	Splicing	Heterozygous	C	T	47.8	1000	novel	Unknown
STX11-exon2	c.^a^ 70G > A	non-coding region	Heterozygous	G	A	53.7	500	rs3734228	0.1166

^a^Accession number of known variant in dbSNP (https://www.ncbi.nlm.nih.gov/projects/SNP/) was listed, others were marked as novel

**Fig. 2:**
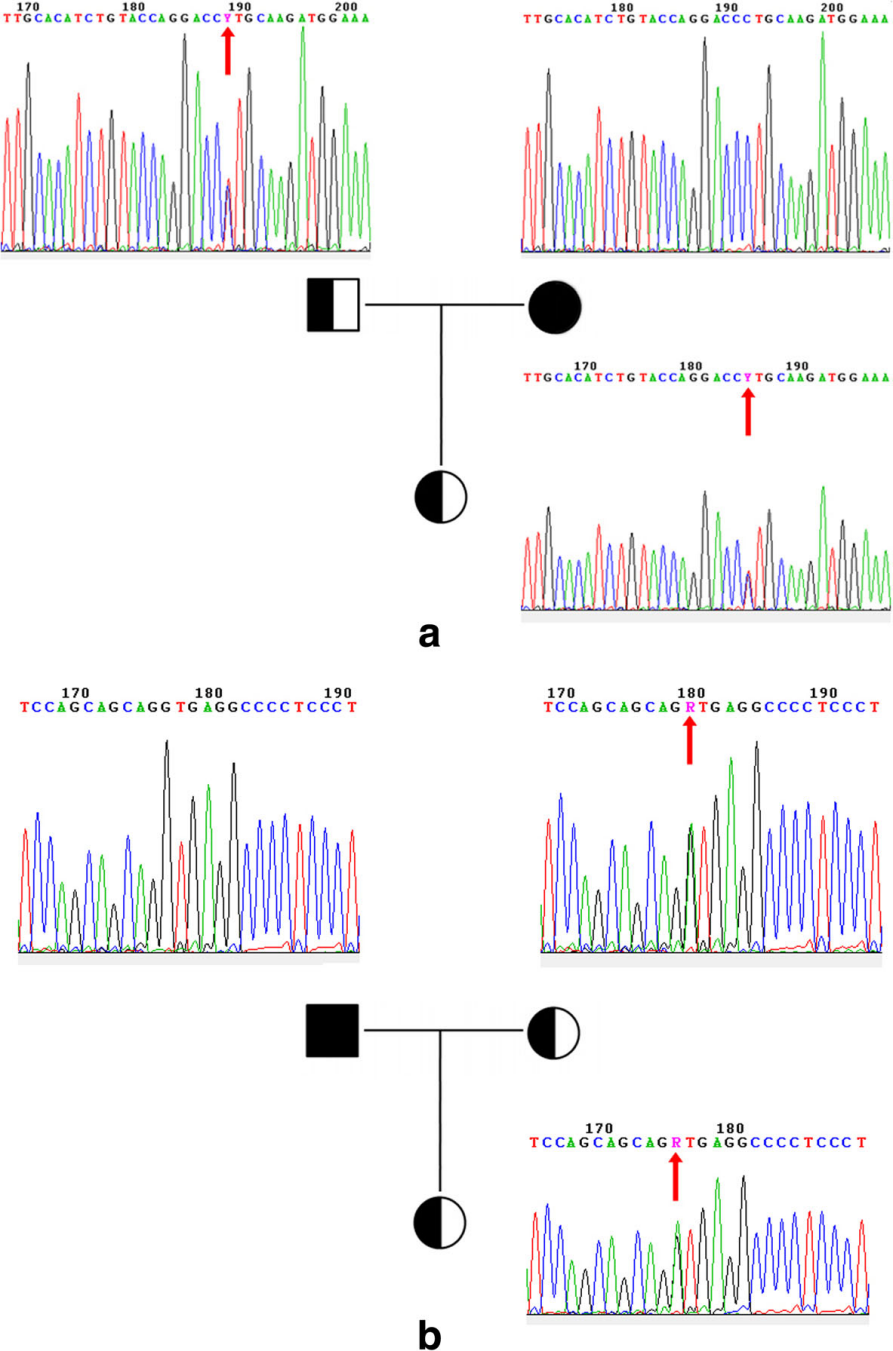
Mutations in UNC13D gene. **a** novel splicing mutation (c.1299-1G > A) in UNC13D-exon15, showed with sequences of the complementary strand. **b** reported splicing mutation (c.2709 + 1G > A) in UNC13D-exon28. Sanger sequencing results of the 18-years-old female patient (underside), her father (top left) and her mother (top right). Mutation position is marked with red arrow. The results show the former was inherited from father while mother has a wild-type locus. In contrast, the latter was inherited from mother, who has a same heterozygous mutation on the locus

Two mutations in *UNC13D* gene were confirmed by Sanger sequencing, analysis for parents showed these two mutations were inherited from father and mother, respectively (Fig. 2). *UNC13D* gene encodes protein that involved in cytotoxic activity of T lymphocytes [10], and it was reported as the predominant causative gene with recurrent splicing mutations in Korean patients with FHL [11]. One splicing mutation in *UNC13D* intron 28 (c.2709 + 1G > A) had been reported in one 9-month-old female [8], while another in exon 15 (c.1299-1G > A) is novel. Though both of them were heterozygous, precedent had shown that compound heterozygous mutations in *UNC13D* gene could be detrimental in FHL [8, 11, 12].

To investigate the functional alteration caused by two splicing mutations in *UNC13D* gene, we performed prediction using the human splicing finder [13]. The result showed that both mutations were most probably affecting splicing, by breaking the wild-type splicing acceptor and donor site, respectively (Additional file 1: Table S1).

## Discussion & conclusions

In this case, an 18-year-old female patient who had high fever for more than 2 days was admitted to Wuhan Children’s Hospital. Hepatomegaly was detected by ultrasound examination. Blood analysis and liver function test showed significant decrease of platelets and NK-cell activity, along with high level of AST, ALT and TG. Hemophagocytosis was observed in bone marrow examination with no evidence of malignancy. All clinical manifestation suggested HLH based on HLH-2004 guidelines. With the confirmation of genetic test, this patient was immediately treated with a full course of etoposide, dexamethasone, cyclosporine A and IVIG, combined with anti-infective therapy and liver protection. The clinical manifestation was quickly stabilized.

Two splicing mutations in *UNC13D* gene that might be pathogenic were identified by amplicon sequencing and confirmed by sanger sequencing. Amplicon sequencing is a rapid and cost-saving approach for detecting mutations in multiple genes, however, the limitations are also obvious. First, most current panels for amplicon sequencing were designed for exons, which led to no coverage of introns and regulatory regions. Second, amplicon sequencing was not capable of detecting mutations in novel genes that might be involved in the disease. These two problems could be solved by whole genome sequencing which is more expensive and time-consuming. Hence, how to choose the appropriate method for genetic testing is worth considering for clinicians and genetic counselors.

*UNC13D* gene encodes protein that involved in cytotoxic activity of T lymphocytes, 17–19% of FHL patients from Turkey and Germany, 89% from Korea and 30% from Japan were identified with *UNC13D* mutations, respectively [11, 14, 15]. Splicing mutations in *UNC13D* gene had been reported as frequent mutations in patients with familial hemophagocytic lymphohistiocytosis type 3 (FHL3). In our patient, a mRNA sequence analysis is required for a further proof. However, FHL is an autosomal recessive disorder, which means children with heterozygous mutations in *UNC13D* gene may have no clinical manifestations. FHL patients with heterozygous mutations could possibly be triggered by external factors such as Epstein-barr virus infection. The susceptibility to EBV infection for different genetic background remaining unrevealed, more statistical data and genetic information is necessary to address this problem.

Rapid and accurate classification for HLH is significant for appropriate treatment, however, subtypes of HLH are difficult to be distinguished from each other clinically. With the help of amplicon sequencing technology, it is possible to detect all mutations in target region in no more than 1 day. Besides, this technology could identify not only known mutations but also novel ones, will greatly improve our understanding about genetic disorders.

In summary, 6 HLH-related genes were tested at once in a suspected HLH patient, two heterozygous splicing mutation in *UNC13D* gene was identified with amplicon sequencing and confirmed by Sanger sequencing, including a novel one. Splicing mutations in *UNC13D* gene had been reported as frequent mutations in FHL3, however, the morbidity of FHL related to splicing mutations in *UNC13D* gene in China is unclear. Rapid and accurate definitive diagnosis is extremely important for appropriate treatment and would benefit life-saving and prognosis-improving, amplicon sequencing shows a great potential in diagnosis for rare genetic disorders.

## Additional file

Additional file 1: Table S1. Functional alteration prediction of two splicing mutations in UNC13D gene with HSF3. (PDF 233 kb)
