# Supplementary material for: Characterization of a novel splicing mutation in UNC13D gene through amplicon sequencing: a case report on HLH
Source: BMC Med Genet. 2017 Nov 21;18:135. doi: 10.1186/s12881-017-0489-1 (PMC5696762; doi:10.1186/s12881-017-0489-1)
Supplement: Additional file 1: Table S1. — Functional alteration prediction of two splicing mutations in UNC13D gene with HSF3. (PDF 233 kb) [file 12881_2017_489_MOESM1_ESM.pdf]

Additional file

Table S1. Functional alteration prediction of two splicing mutations in UNC13D gene with HSF3.

<table>
<tr><th>Mutation</th><th>Predicted signal</th><th>Prediction algorithm</th><th>cDNA Position</th><th>Interpretation</th></tr>
<tr><td rowspan="2">c.2709+1G>A</td><td rowspan="2">Broken WT Donor Site</td><td>1 - HSF Matrices</td><td rowspan="2">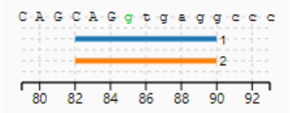
</td><td rowspan="2">Alteration of the WT donor site, most probably affecting splicing.</td></tr>
<tr><td>2 - MaxEnt</td></tr>
<tr><td rowspan="4">c.1299-1G>A</td><td rowspan="2">Broken WT Acceptor Site</td><td>1 - HSF Matrices</td><td rowspan="2">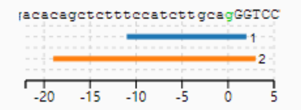
</td><td rowspan="2">Alteration of the WT acceptor site, most probably affecting splicing.</td></tr>
<tr><td>2 - MaxEnt</td></tr>
<tr><td rowspan="2">New Acceptor Site</td><td>1 - HSF Matrices</td><td rowspan="2">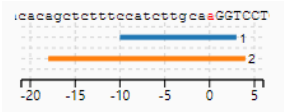
</td><td rowspan="2">Activation of an intronic cryptic acceptor site.
Potential alteration of splicing.</td></tr>
<tr><td>2 - MaxEnt</td></tr>
</table>
